# Cost-effectiveness of micafungin as an alternative to fluconazole empiric treatment of suspected ICU-acquired candidemia among patients with sepsis: a model simulation

**DOI:** 10.1186/cc7924

**Published:** 2009-06-19

**Authors:** Marya D Zilberberg, Smita Kothari, Andrew F Shorr

**Affiliations:** 1 School of Public Health and Health Sciences, University of Massachusetts, Arnold House, 715 North Pleasant Street, Amherst, MA 01003, USA; 2 Evi*Med *Research Group, LLC, PO Box 303, Goshen, MA 01032, USA; 3 Health Economics and Outcomes Research, Astellas Pharma US, Inc., 3 Parkway North, Deerfield, IL 60015, USA; 4 Division of Pulmonary and Critical Care, Washington Hospital Center, 100 Irving Street NW, Washington, DC 20010, USA

## Abstract

**Introduction:**

Recent epidemiologic literature indicates that candidal species resistant to azoles are becoming more prevalent in the face of increasing incidence of hospitalizations with candidemia. Echinocandins, a new class of antifungal agents, are effective against resistant candidal species. As delaying appropriate antifungal coverage leads to increased mortality, we evaluated the cost-effectiveness of 100 mg daily empiric micafungin (MIC) vs. 400 mg daily fluconazole (FLU) for suspected intensive care unit-acquired candidemia (ICU-AC) among septic patients.

**Methods:**

We designed a decision model with inputs from the literature in a hypothetical 1000-patient cohort with suspected ICU-AC treated empirically with either MIC or FLU or no treatment accompanied by a watchful waiting strategy. We examined the differences in the number of survivors, acquisition costs of antifungals, and lifetime costs among survivors in the cohort under each scenario, and calculated cost per quality adjusted life year (QALY). We conducted Monte Carlo simulations and sensitivity analyses to determine the stability of our estimates.

**Results:**

In the base case analysis, assuming ICU-AC attributable mortality of 0.40 and a 52% relative risk reduction in mortality with appropriate timely therapy, compared with FLU (total deaths 31), treatment with MIC (total deaths 27) would result in four fewer deaths at an incremental cost/death averted of $61,446. Similarly, in reference case, incremental cost-effectiveness of MIC over FLU was $34,734 (95% confidence interval $26,312 to $49,209) per QALY. The estimates were most sensitive to the QALY adjustment factor and the risk of candidemia among septic patients.

**Conclusions:**

Given the increasing likelihood of azole resistance among candidal isolates, empiric treatment of ICU-AC with 100 mg daily MIC is a cost-effective alternative to FLU.

## Introduction

Emerging antimicrobial resistance poses a significant threat to health care in the USA and abroad. Pathogens such as methicillin-resistant *Staphylococcus aureu*s, extended-spectrum beta-lactamase Gram-negative organisms, *Clostridium difficile *and *Pseudomonas aeruginosa *are increasingly encountered and now exhibit resistance to some traditional therapies. This same trend has been observed with fungal infections. First, there has been a shift in the distribution of species responsible for candidal bloodstream infections (BSIs). Multiple analyses document that one-third to one-half of all candidal BSIs are due to non-albicans species [1-5]. Second, and more importantly, *Candida glabrata *(CG) and *Candida krusei *(CK) now account for as many as 20 to 25% of all candidal BSIs [6-9]. These two specific pathogens are generally not susceptible to fluconazole, a drug commonly used in critically ill patients.

Accompanying these microbiologic and epidemiologic shifts, the burden of candidal BSIs is rising. The past six years has witnessed a 50% growth in hospitalizations complicated by candidal BSIs [10]. This trend has also been seen in critically ill patients [11]. Unfortunately, many clinicians do not consider fungi as a cause of severe sepsis and BSIs. This is of particular concern given that either a delay in initiation of antifungal therapy or treatment with an antifungal that is not active against the specific fungal pathogen independently raises a patient's risk of death [12,13].

To address the need to provide initially appropriate antibacterial therapy, many advocate a strategy based on de-escalation. In this paradigm, one begins treatment with multiple agents or agents with broader coverage profiles based on local susceptibility data and then adjusts and/or discontinues anti-infectives as culture and sensitivity (C&S) results return. One concern about a de-escalation approach to potential fungal BSIs is that echinocandins, which represent a commonly used class of broad-spectrum antifungal agents used in the intensive care unit (ICU), are expensive relative to the burden of Candida. In essence the issue becomes, in part, that of the cost and benefit balance.

Therefore, because ICU patients with hospital-acquired sepsis represent a group at high risk for the development of ICU-acquired candidemia (ICU-AC) [14-17], and given the increasing frequency of resistant candidal species coupled with the clinical importance of broad empiric coverage, we hypothesized that empiric utilization of the newly-approved dosage of 100 mg daily of micafungin (MIC) [18], an echinocandin, would be a cost-effective strategy when compared with either empiric fluconazole (FLU) treatment or to a strategy of watchful waiting pending C&S results. To test this hypothesis we developed a decision model to quantify cost and outcome differences between these various options.

## Materials and methods

No human subjects were enrolled in the study, and, thus, the study was exempt from regulations guiding protection of human subjects. This is an analysis of publicly available data. All calculations were performed in Microsoft Excel 2003 (Microsoft, Redmond, WA, USA). Multivariate simulations and sensitivity analyses were performed using Crystal Ball software (Decisioneering, Denver, CO, USA).

### Model overview and structure

**Figure 1:**
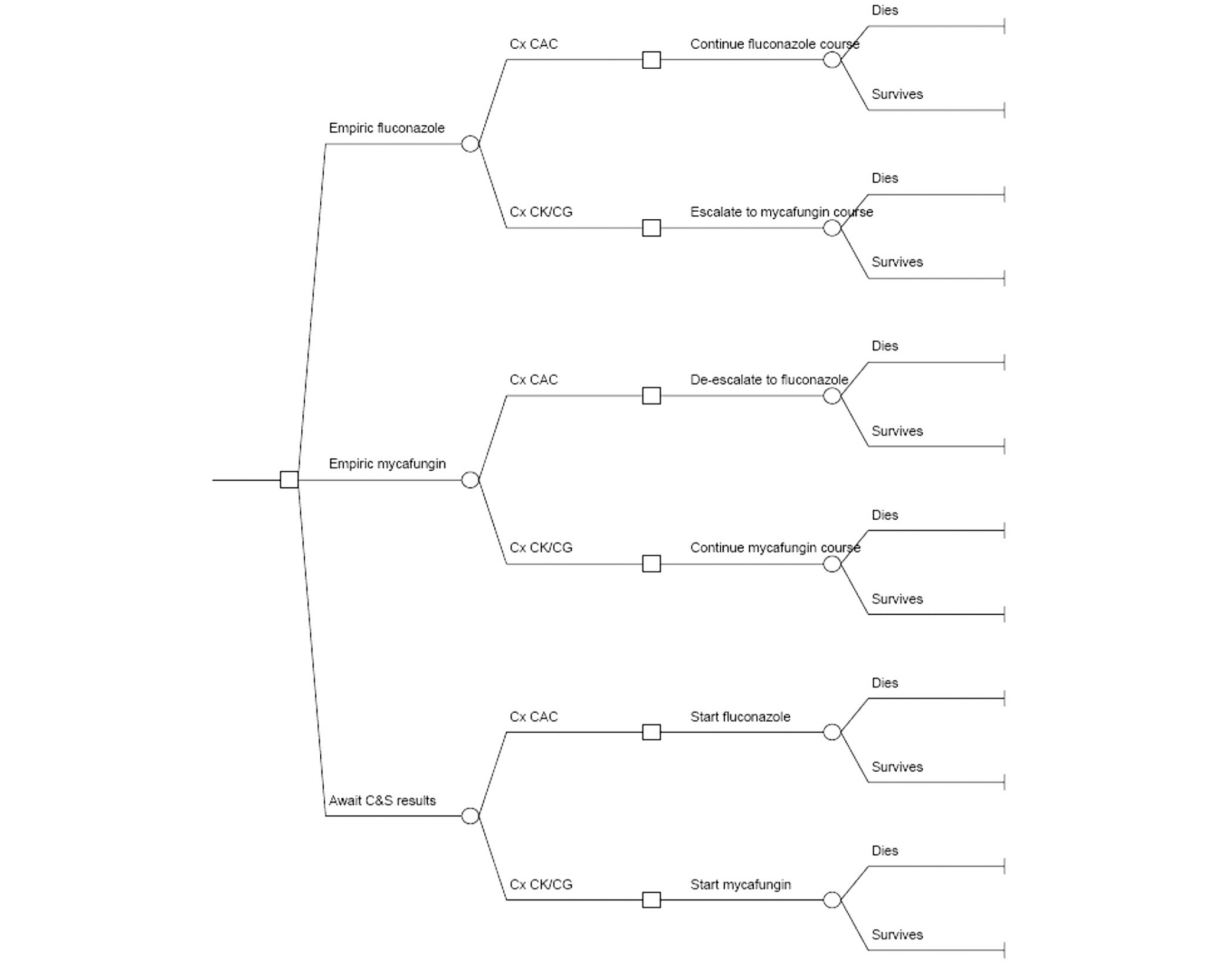
Decision tree. The square node at the left of the diagram represents the decision to treat with micafungin or fluconazole empirically or to adopt the 'await culture and sensitivity (C&S) results' watchful waiting strategy prior to instituting treatment. The circular chance nodes represent the probability of the blood culture specimen returning *Candida albicans *(CAC) vs. *C. glabrata *(CG) or *C. krusei *(CK). The second square decision node represents the decision made about antifungal therapy in response to the C&S data, and the final circular event node represents the outcome of death vs. survival. The right most vertical segments are terminal nodes.

We developed a cost-effectiveness model comparing MIC with FLU and MIC or FLU with watchful waiting as the empiric treatment for suspected ICU-AC. We performed our analyses from the perspectives of both the hospital and population in general. We followed the recommendations of the Panel on Cost-Effectiveness in Health and Medicine [19]. We utilized a decision-analysis approach with a single decision node representing the choice to institute treatment with MIC or FLU, or to withhold treatment until culture confirmation. We conducted an analysis in a hypothetical cohort of 1000 patients in the ICU. We marched this cohort through each branch of the decision tree separately to either death or illness resolution and calculated the incremental costs associated with each choice (Figure 1). The parameters for the incidence of candidemia overall and the likelihood of infection with resistant species (CG or CK), and the various clinical and economic consequences were derived from a review of pertinent literature and other publicly available data (Table 1). We examined the differences in hospital mortality, drug acquisition costs, and total lifetime health care costs in the cohort under each scenario. Because our literature review failed to identify any studies quantifying the incremental utilization and economic outcomes associated with inappropriate empiric treatment of candidemia, and to avoid introducing additional uncertainty into the model by using unsupported estimates, we were unable to include this component of health care costs into our calculations. However, given the recent report that direct variable hospital costs are minimally impacted by reducing ICU or hospital length of stay, the current inputs are likely to be of greatest relevance [20]. Therefore, we defined cost-effectiveness from the hospital perspective as the ratio between drug acquisition cost differences and the differences in the numbers of hospital deaths between the strategies. In turn, the reference case built from the societal perspective examined lifetime health care costs of survivors per quality-adjusted life year (QALY) as the unit of cost-effectiveness of each strategy.

**Table 1:** Model inputs

**Parameter**	**Point estimate**	**Range**	**Source**
*Candidemia risk and mortality*			
Risk of candidemia	0.140	0.048 to 0.283	See Table S1 in Additional data file 1
Risk of CK/CG among candidemia	0.148	0.132 to 0.361	See Table S2 in Additional data file 1
Candidemia attributable mortality	0.4	0.2 to 0.8	Golan and colleagues [22]
Mortality reduction with appropriate empiric therapy	0.48	0.35 to 0.65	Morrell and colleagues [12] (inverse)
*Evaluation and treatment*			
Days of empiric treatment until availability of C&S results	3	2 to 4	Assumption
Total duration of appropriate treatment (days)	10	7 to 14	Guideline recommendations of 14 days reduced to 10 days to account for mortalities
Days of antibiotic treatment if switched from MIC to FLU in response to C&S	7	4 to 10	Total duration of appropriate treatment – Days of empiric treatment until availability of C&S results
Days of antibiotic treatment if switched from FLU to MIC in response to C&S	10	7 to 14	Total duration of appropriate treatment
*Cost of antibiotics*			
MIC ($/100 mg daily IV)	$100	$80 to 120	WHC Pharmacy
FLU ($/400 mg daily IV)	$12	$10 to 14	WHC Pharmacy
FLU ($/400 mg single loading dose on day 1)	$12	$10 to 14	WHC Pharmacy
*Life expectancy, QALY adjustment and lifetime costs*			
Median age (years)	64	48 to 80	Median from Table S1 in Additional data file 1; range is +/- 25% median
Life expectancy (years)	17.4	13.0 to 21.4	Actuarial tables from the US Social Security Administration [24]; for men; range is +/-25%
Relative risk of death	0.51	0.49 to 0.59	Quartin and colleagues [25]
QALY adjustment	0.64	0.44 to 0.80	Fowler and colleagues [26], and Davies and colleagues [27]
Age-specific annual healthcare costs/survivor in 2001 $US*	$16,446	$12,335 to $20,558	Shorr and colleagues [29]
Annual discount rate	3%	0 to 6%	Weinstein and colleagues [19]

*Inflating to 2008 $US using the medical component of the consumer price index resulted in $22,080, 95% confidence interval $16,588 to $27,601.

CG = *Candida glabrata*; CK = *Candida krusei*; C&S = culture and sensitivity; FLU = fluconazole; iv = intravenous; MIC = micafungin; QALY = quality-adjusted life year.

WHC = Washington Hospital Center, Washington, DC, USA

Because of the uncertainty surrounding the point estimates for various model inputs, we conducted several Monte Carlo simulations and sensitivity analyses to assess the precision and stability of these observations. The 95% confidence intervals (CIs) around the point estimates were calculated based on the Monte Carlo simulations. One-way sensitivity analyses were performed to establish cost-effectiveness threshold values and the worst-case scenario. Two-way sensitivity analyses were conducted to establish the ranges in the outcome estimate introduced by two of the inputs contributing most to the uncertainty in the model.

### Model inputs

#### Incidence of ICU-acquired candidemia

For the proportion of patients in the ICU developing candidemia, we derived numbers from several large international cohort studies. Although we initially set out to build a model specifically relevant to the USA, our search did not identify any generalizable studies in this area. Looking to the studies outside the USA, four reported in various ways the prevalence of ICU-AC as it applies to the ICU population of patients with sepsis [14-17]. The four studies all represent recent epidemiologic cohorts in sepsis populations [see table S1 in additional data file 1]. Based on these four studies, we used the mean prevalence of candidal culture positivity as the point estimate and the range as the uncertainty range around the point estimate.

#### Incidence of resistant candidal species

We examined the literature from the US institutions published in the current decade pertaining to the setting of the adult ICU. Based on the six studies so identified [4,6-9,21] [see table S2 Additional data file 1], we calculated the pooled prevalence of CK and CG as a function of all positive candidal blood cultures and used this number as the point estimate, and the range of prevalences from the six studies as the range around the point estimate. Despite the fact that not all CG and CK isolates are azole resistant, we essentially used CG/CK as a surrogate for azole resistance for two reasons. One practical consideration for this choice was driven by non-availability at some centers of data either on the species of Candida (other than a report that the isolate is non-albicans) or the minimum inhibitory concentrations of FLU for the species isolated. In the first case, unless no FLU resistance is present locally among any CG isolates, a clinician may be forced to generalize that all non-albicans species be treated as if they were FLU resistant. Similarly, in the case of non-availability of data on minimum inhibitory concentrations of FLU, a clinician may be unable to differentiate between the need to dose escalate or switch to a broader-spectrum agent. To make our model as applicable as possible to these real-world situations, and because a multicenter study indicated that the overall rate of FLU resistant candidal species is approximately 10% [6], we performed multiple additional threshold analyses to explore how further reducing the prevalence of CK/CG (as the marker for azole resistance) from our minimum estimates would affect the cost-effectiveness profiles of our strategies.

#### Attributable mortality and mortality reduction with appropriate treatment

We used the attributable mortality for untreated candidemia as that reported in the recent study by Golan and colleagues [22]. We estimated the mortality reduction conferred by timely institution of an appropriate therapy based on a study by Morrell and colleagues [12]. In this study the investigators noted a 2.09-fold (95% CI = 1.53 to 2.84) increase in the risk of hospital death associated with a delayed institution of antifungal therapy among critically ill patients with candidemia [12]. To arrive at the reduction in attributable mortality due to prompt empiric therapy of ICU-AC, the odds ratio, and the corresponding 95% CI were inverted.

#### Duration, dosage and costs of antifungal treatment

Duration and dosage of FLU treatment were based on the guidelines of the Infectious Diseases Society of America [23]. A 10-day treatment course was substituted in the base case for the recommended 14 days as an approximate adjustment for the fact that a certain percentage of treated patients will die prior to completing their FLU course. The duration and dosage of MIC were based on the label recommendation for this drug with a similar adjustment for early mortality [18]. The duration of treatment was varied between 7 and 14 days. FLU was to be administered daily at the dose of 400 mg intravenously, with an additional 400 mg loading dose on day 1, while MIC was assumed to be given at 100 mg intravenously daily. In the base case a three-day lag period between the onset of ICU-AC and the availability of C&S results was assumed, and this was varied between two and four days. Patients treated with MIC who tested positive for *Candida albicans *(CA) were switched to FLU and received an additional seven days of treatment for a complete 10-day course, while those treated with FLU empirically and testing positive for CG or CK were switched to MIC for an additional 10-day course of appropriate coverage. This allowed us to capture the potential for 'step-down' therapy.

The drug acquisition costs for both MIC and FLU were obtained from the pharmacy department of one of the authors' (AFS) institution. For the treatment duration and costs we chose to use conservative estimates erring on the side of biasing our results against MIC.

#### Life expectancy, risk of death and QALY adjustment

The median age of the ICU-AC patients was derived from the four studies providing epidemiologic data on ICU candidemia [14-17], with the base case patient being 64 years of age, and the range for sensitivity analyses of +/- 25% of the base case. We obtained life expectancy from the actuarial tables provided by the US Social Security Administration [24], and this number was varied by 25% in the sensitivity analyses. The risk of post-hospitalization death was extracted from a study of septic patients by Quartin and colleagues [25], and the product of this value and the life tables life expectancy served as the estimate of life expectancy of a sepsis survivor. The QALY adjustment was based on the mean of those reported by Fowler and colleagues [26] and Davies and colleagues [27]. Age-specific annual health care costs/survivor reported by Shorr and colleagues [28] in 2001 $US were inflated to 2008 $US using the medical component of the consumer price index and varied across the corresponding 95% CI [28,29]. All projected costs were discounted at 3% per annum (range 0 to 6%), as recommended by the Panel on Cost-Effectiveness in Health and Medicine [19].

## Results

In the base case scenario, among the 1000 patients there were 140 cases of ICU-AC, of which 21 represented CG or CK. Compared with the cohort treated with empiric FLU, treatment with MIC resulted in four (95% CI = 2 to 13) additional deaths averted at the marginal drug acquisition cost of $61,446 (95% CI = $43,821 to $80,039) per life saved (Table 2) and the total incremental outlay for empiric antifungal treatment of $245,784 over FLU. Taking into account life expectancy of the survivors and factoring in the QALY adjustments, this translated to $22,230 (95% CI = $18,201 to $26,088) per life year saved and $34,734 (95% CI = $26,312 to $49,209) per QALY (Table 2). Compared with the watchful waiting strategy, either of the treatment scenarios generated substantially more survivors (29 MIC (95% CI = 11 to 69) and 25 FLU (95% CI = 9 to 57)) at between $1704 (95% CI = $640 to 4839 FLU) and $9982 (95% CI = $3771 to $26,065 MIC) per death averted (Table 2). The cost per life year saved and that per QALY were similar for MIC and FLU when compared with watchful waiting (Table 2).

**Table 2:** Outcomes per cohort of 1000 critically ill patients with suspected nosocomial candidemia

**Outcome**	**Point estimate**	95% confidence interval*
*Empiric MIC compared with empiric FLU*		
Deaths averted	4	2 to 13
Incremental cost/death averted^†^	$61,446	$43,821 to $80,039
Incremental cost/life year saved^†¶^	$22,230	$18,201 to $26,088
Incremental cost/QALY^†¶^	$34,734	$26,312 to $49,209
*Empiric MIC compared with watchful waiting strategy*		
Deaths averted	29	11 to 69
Incremental cost/death averted^†^	$9,892	$3,771 to $26,065
Incremental cost/life year saved^†¶^	$17,777	$14,174 to $21,360
Incremental cost/QALY^†¶^	$27,777	$20,572 to $39,888
*Empiric FLU compared with watchful waiting strategy*		
Deaths averted	25	9 to 57
Incremental cost/death averted^†^	$1,704	$640 to $4,839
Incremental cost/life year saved^†¶^	$17,070	$13,582 to $20,436
Incremental cost/QALY^†¶^	$26,672	$19,699 to $38,223

*From the Monte Carlo simulations, 10,000 trials for each outcome.

^†^All costs inflated to 2008 $US using medical component of the consumer price index.

^¶^Costs incorporating lifetime estimates are discounted at 3% per annum (range 0 to 6%)

FLU = fluconazole; MIC = micafungin; QALY = quality-adjusted life year.

**Figure 2:**
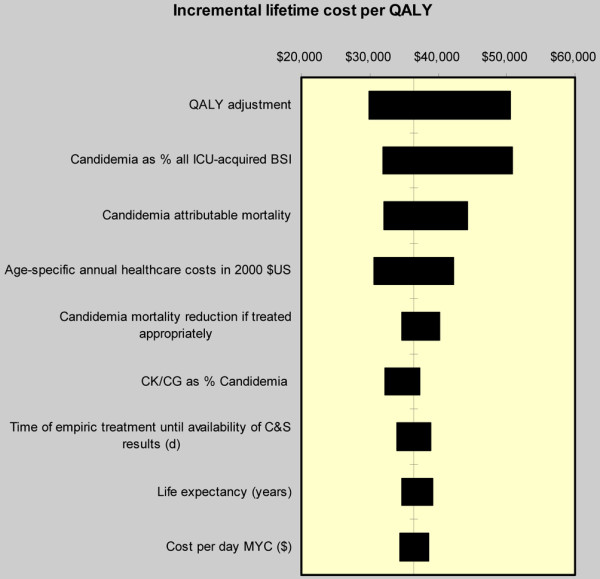
Tornado diagram. The solid vertical line represents the total incremental lifetime costs per quality-adjusted life year (QALY) for survivors using micafungin compared with fluconazole as empiric treatment of suspected intensive care unit (ICU)-acquired candidemia as calculated in the base case scenario. The horizontal bars represent the range of this difference when the corresponding single input is varied across its designated range with all other input parameters held constant. All costs inflated to 2008 $US using medical component of the consumer product index; a 3% annual discount applied. BSI = blood stream infection; C&S = culture and sensitivity; CG = *Candida glabrata*; CK = *Candida krusei*, MYC = micafungin.

Each of the models assessing incremental costs per QALY was most sensitive to the QALYs generated by survivors (Figure 2, and data not shown). In a univariate analysis varying the number of QALYs gained (0.44 to 0.80) resulted in the range of cost/QALY of $21,337 to $38,795 in the case of FLU and $22,221 to $40,402 for MIC each vs. the watchful waiting strategy, and $27,310 to $49,655 for MIC vs. FLU. The MIC vs. FLU analysis was also sensitive to the proportion of all BSIs attributed to Candida species (Figure 2), and varying this single input across its corresponding range resulted in a cost/QALY range of $30,151 to $49,262. Even if the empiric MIC strategy results in only one additional life saved in the 1000-patient cohort compared with FLU, the cost/QALY remained $59,610.

Table 3 presents the results of the two-way sensitivity analysis simultaneously varying the two inputs with the most impact on the variability of the MIC vs. FLU model – the QALY adjustment factor and the proportion of BSI attributed to Candida species. This analysis confirms the durability of the cost-effectiveness ratio of the MIC vs. FLU empiric treatment strategy in this population. Even in the event of extremely low prevalence of candidemia (0.048) combined with a large decrement in the remaining quality of life, the cost remains $50,722 per QALY. In the worst-case analysis, with all of the inputs skewed maximally against MIC, the incremental cost per QALY is $72,318 (Table 4). In the final threshold analysis where we progressively reduced the CG/CK isolates as a proportion of all ICU-AC, the incremental lifetime costs per QALY for the MIC vs. FLU strategy were just under $50,000 ($49,789) for CG/CK prevalence of 5%, and went over the $100,000 per QALY threshold at the prevalence of 1.5% ($104,490).

**Table 3:** Two-way sensitivity analysis: impact of simultaneously varying the QALY adjustment and proportion of BSI represented by Candida on the estimate of cost per QALY of MIC vs. FLU

**QALY conversion factor**	**Candidemia as percentage of all BSI**					
	**0.048**	**0.100**	**0.140**	**0.200**	**0.250**	**0.283**
**0.44**	$50,722	$50,609	$50,522	$50,391	$50,282	$50,210
**0.50**	$44,636	$44,536	$44,459	$44,344	$44,248	$44,185
**0.55**	$40,578	$40,487	$40,417	$40,313	$40,226	$40,168
**0.60**	$37,196	$37,113	$37,049	$36,953	$36,874	$36,821
**0.64**	$34,872	$34,794	$34,734	$34,644	$34,569	$34,520
**0.70**	$31,883	$31,811	$31,757	$31,674	$31,606	$31,561
**0.75**	$29,757	$29,691	$26,639	$29,563	$29,499	$29,457
**0.80**	$21,897	$27,835	$27,787	$27,715	$27,655	$27,616

BSI = blood stream infection; FLU = fluconazole; MIC = micafungin; QALY = quality-adjusted life year.

**Table 4:** Worst case scenario, with all inputs biased against the empiric MIC vs. FLU strategy

**Input**	
Candidemia as % BSI	0.48
CK/CG as % candidemia	0.132
Candidemia attributable mortality	0.2
Candidemia mortality reduction if treated appropriately	0.35
Total duration of treatment with appropriate treatment (days)	14
Time to C&S results (days)	4
Time initial treatment following C&S results (days)	10
Time treatment following switch for C&S results (days)	14
Cost per day FLU ($)	$10
Cost per extra 400 mg loading day 1 in FLU gr	$10
Cost per day MIC ($)	$120
Age	80
Life expectancy	13
Relative risk of death	0.59
Time-trade off utility (QALY adjustment)	0.44
Age-specific annual healthcare costs in 2001 $US*	$20,558
Annual discount rate	0%

**Outcome**	

Incremental lifetime cost per QALY	$72,318

*All costs inflated to 2008 $US using medical component of the consumer price index.

BSI = bloodstream infection; CG = *Candida glabrata*; CK = *Candida krusei*; C&S = culture and sensitivity; FLU = fluconazole; MIC = micafungin; QALY = quality-adjusted life year.

## Discussion

The cost-effectiveness analysis demonstrates that in patients with ICU-acquired sepsis who are at risk of candidemia, a strategy of empiric treatment with MIC is cost-effective. Compared with either an approach of watchful waiting and delaying antifungal therapy until the return of cultures, or one of empiric FLU use rather than treatment with an echinocandin, a de-escalation approach to candidemia enhances survival. More importantly, this improvement in outcomes comes at a modest cost from either a hospital or societal perspective. The fact that our point estimates for the cost-effectiveness of MIC as initial empiric therapy are insensitive to multiple input variables confirms the robustness of our observations.

With respect to traditional bacterial pathogens, experts advocate de-escalation as the only means for ensuring that a patient receives initial antibiotic therapy in an era of growing resistance. Furthermore, de-escalation emphasizes the need to narrow the spectrum of antibiotic treatment as culture data become available in order to help prevent antibiotic abuse and the spread of new resistance. In general, a de-escalation approach is thought to be cost-effective [30]. Due to a combination of the prevalence of bacterial resistance, the limited costs of several days of even broad-spectrum antibiotics, and the attributable mortality related to inappropriate initial therapy [12,13,22,31-36], de-escalation represents a potential means for enhancing outcomes with limited additional pharmacy outlays. Moreover, inappropriate antibiotic treatment itself has been shown to increase both length of stay and hospital costs independent of its impact on mortality [37]. Our analysis builds on earlier work dealing with the economics of de-escalation in bacterial infections [37]. We confirm that the cost-effectiveness of de-escalation extends beyond purely bacterial infections. Thus, this approach can be more generally applied to cohorts of critically ill patients developing hospital-acquired fungal sepsis.

A recent study by Schuster and colleagues examined the effectiveness of presumptive antifungal treatment in ICU patients developing fever while receiving antibiotics [38]. In this randomized controlled trial, broad empiric use of FLU did not impact the outcomes. Although the population of patients in this trial may seemingly resemble the patients examined in our model, there are major differences between them. Thus, the population of interest in our model is ICU patients who develop hospital-acquired sepsis, as signified not only by fever, but by signs of hemodynamic instability and organ failure. In contrast, Schuster and colleagues enrolled patients with fever but not necessarily with other signs of sepsis [38]. Additionally, the rates of proven candidemia in this trial were exceedingly low (5 to 9%), much lower than those in the population we examined in our model (14% in the base case and from 5% at the lowest end up to 48% as the upper limit; Table 1). For all these reasons, the results of the trial by Schuster and colleagues cannot be generalized to the population examined in the current study.

One other study has examined the cost-effectiveness of empiric antifungal treatment among ICU patients with suspected candidemia [22]. In a decision model, Golan and colleagues examined the effect of nine clinical strategies, including four empiric and four culture-based treatment choices, as well as one no-treatment strategy, in a cohort of persons at increased risk for Candida BSI [22]. In this simulation, utilizing some of the inputs similar to ours, the authors found caspofungin to be most effective, but prohibitively expensive ($295,115 per discounted life-year saved), and empiric FLU treatment to be the 'most reasonable' strategy, costing $12,593 per discounted life-year saved. Our modeling approach and observations add further to the efforts made by Golan and colleagues [22]. For example, we examined a newer echinocandin, MIC, which, at the dose we utilized in our model, has substantially lower acquisition costs than caspofungin did at the time Golan and colleagues completed their project. Additionally, and of interest to intensivists, we provided information on a very select group of subjects who by their very nature face a higher risk of candidemia than do patients in the mixed population evaluated by Golan and colleagues [22]. Finally, we quantified the differences in outcomes and costs between either MIC or FLU and watchful waiting. By finding a cost-effectiveness ratio of $34,734 per QALY for MIC vs. FLU, as well as very similar cost-effectiveness profiles for both MIC and FLU compared with watchful waiting, we further justify and focus the attention to clinical effectiveness outcomes in this population of critically ill patients at risk. In that same vein, given the predicted growing burden of candidemia as a cause of severe sepsis along with the rising prevalence of CK and CG, our results are likely to hold true for the future.

Interestingly, our observations were sensitive to two specific variables – adjustments for QALY after surviving sepsis and the incidence of sepsis due to candidemia. In one sense it is not surprising that the resulting number of QALYs affected the model's output, because this represented the denominator in one of our outcome measures. The finding that varying the proportion of nosocomial sepsis due to candidemia may alter the cost-effectiveness of the various strategies evaluated suggests that clinicians need to be aware of the burden of Candida in their own institutions. However, when we modeled a relatively low rate of candidemia, empiric treatment with MIC remained a cost-effective approach. Furthermore, the model was less affected by either the proportion of candidemia due to CK or CG or the attributable mortality related to inappropriate therapy. In fact, even in the setting of lower than generally reported prevalence of azole-resistant candidal isolates, this strategy remains cost-effective. This potentially indicates that the major issue for clinicians in terms of outcomes is less the failure to consider non-albicans species of yeast, but more broadly failing to place candidemia in the differential diagnosis for nosocomial sepsis in a critically ill patient.

Because national healthcare expenditures in the USA have seen unparalleled growth, and hospital care accounts for one-third of the total national healthcare expenditures [39], patients, payers, and policy makers are paying close attention to how these resources are allocated. In the era of fiscal restraint, potentially difficult choices need to be made that necessarily limit utilization of resources. To make these choices, it is helpful to have data pertaining not only to efficacy and effectiveness of a given therapy, but also to its cost-effectiveness. In order to compare across unrelated therapeutic areas and to make the best value choices, a cost-effectiveness analysis must provide the reference case. In our model we calculated the cost-effectiveness in the reference case to be $34,734 per QALY, a number well below the traditional cost-effectiveness threshold of $100,000. In this sense, empiric MIC treatment compares favorably to other therapies utilized in the ICU. In a recent systematic review Talmor and colleagues identified 50 cost-effectiveness studies in the peer-reviewed literature pertaining to the ICU setting [40]. In this review they noted that there is a "general consensus that treatments with a cost-effectiveness ratio of $50,000 to $100,000 per year of life gained are acceptable in the United States today" [40]. Their findings suggested that the cost-effectiveness ratios observed, ranging from cost-saving to nearly $1 million per QALY, were exquisitely sensitive to patient characteristics and case mix. As our study focused on a rather narrow population of the critically ill patients, the case-mix is not likely to affect our estimates to any significant degree.

Our study has a number of limitations. We derived our drug acquisition cost estimates from a single institution, and, as such, the numbers herein may not be generalizable to all US institutions. On the other hand, we chose to use this input because it provides a more bona fide reflection of the real-world drug acquisition costs than either the average wholesale price or the wholesale acquisition cost. To improve the generalizability of this parameter estimate, we varied this input across a reasonable range. Although the inputs for the prevalence of ICU-AC are derived from four large multi-center cohort studies of patients with sepsis, we were unable to validate these data in the US setting. Additionally, the estimates of CG and CK as a percentage of all candidemia varied widely from 13% [21] to 36% [9], implying that local resistance patterns are critical to apply to get accurate model estimates. To circumvent this imprecision, however, we tested a wide range of values for this observation in our model, and, despite its moderate contribution to the precision of the model (Figure 2), the cost-effectiveness of MIC over FLU persisted. Another important limitation of the current study is our inability to quantify the contribution of inappropriate anti-fungal therapy to resource utilization and hospital costs, because reliable estimates for these parameters are simply not available in the literature. For this reason, we relied solely on drug acquisition costs to differentiate these outcomes between the strategies. However, it has been estimated that only 14% of all hospital costs are due to the variable component [41], and more recent work quantifies the cost differential between the last ICU and first ward day to be $118, while the direct variable cost of a ward day is only $109 [20]. These data afford some assurance that the actual total cost estimates would not be affected dramatically by including or excluding these costs. However, there is little reason to believe that, by not quantifying the contributions to the alterations in the hospital length of stay and the consequent throughput issues, we have biased the model in favor of MIC, because in other infections inappropriate empiric treatment is associated not only with heightened mortality, but also with an increase in the length of stay and costs [37]. If this is the case for candidemia, then our model may in fact underestimate the cost-effectiveness of broad empiric coverage. Another important consideration warrants discussion. We note that only 14% (range 5% to 28%) of the patients suspected of having ICU-AC actually have candidemia, leaving the remaining 86% with several days of empiric broad-spectrum antifungal exposure. Therefore, as with any condition in which the threat of resistance is high, the benefit of empiric MIC treatment of all patients suspected of having ICU-AC must be balanced against the risk of overtreatment and the resultant development of resistance. As a de-escalation approach is the recommended solution to this dilemma, we have modeled precisely such a strategy.

## Conclusions

In summary, our model suggests that empiric treatment of septic ICU patients with suspected ICU-AC with MIC is cost-effective compared with treatment with FLU at the threshold below the $50,000 to $100,000 per QALY. Furthermore, MIC exhibits a similar cost-effectiveness profile to FLU when each is compared with delaying treatment pending culture results. As the concerns for emergence of resistant Candida species heighten in the US health care institutions, clinicians at the bedside need to make targeted empiric treatment choices. Although our study indicates that starting treatment empirically with MIC in the population described is a cost-effective alternative, such broad coverage could promote selection of echinocandin-resistant species. To prevent resistance, current practice dictates prompt de-escalation of therapy in response to culture results. Knowing the local resistance patterns is also critical for every clinician making empiric treatment choices. Finally, risk stratification algorithms identifying the profiles of patients likely to be infected with resistant Candida species could improve the precision of antibiotic selection in this population with the view to not only improving individual patient outcomes, but also preserving the effectiveness of a limited health care resource.

## Key messages

• Azole-resistant candidal species are growing in prevalence.

• Empiric coverage with an antifungal agent to which the isolates are sensitive may improve outcomes.

• Treatment of suspected ICU-AC with the echinocandin MIC at a dose of 100 mg daily is a cost-effective alternative compared with 400 mg daily FLU or observation alone.

## Abbreviations

BSI: bloodstream infections; CA: *Candida albicans*; CG: *Candida glabrata*; CI: confidence interval; CK: *Candida krusei*; C&S: culture and sensitivity; FLU: fluconazole; ICU: intensive care unit; ICU-AC: ICU-acquired candidemia; MIC: micafungin; QALY: quality-adjusted life year.

## Competing interests

This study was funded by a grant from Astellas Pharma US Inc, the manufacturer of micafungin. MDZ and AFS are consultants to and SK is an employee of Astellas. SK is a stock holder in Astellas Pharma US, Inc, the manufacturer of micafungin. AFS and MDZ have received research and consulting funding, and SK receives a salary from Astellas Pharma US Inc, the manufacturer of micafungin. The funding body did not participate in the study design or data collection. The authors made all publication decisions.

## Authors' contributions

MDZ participated in the design, interpretation and drafting of the study, and carried out all of the analyses for the study. SK participated in the interpretation and drafting of the study. AFS participated in the design, interpretation, and drafting of the study.

## Supplementary Material

Additional data file 1 **Table S1 lists the input sources for estimating the prevalence of nosocomial candidemia in critically ill patients**. Table S2 lists the input sources for estimating the prevalence of *Candida krusei *and *Candida glabrata *as a proportion of all nosocomial candidemia in critically ill patients. Click here for file
