# Supplementary material for: Cost-effectiveness of micafungin as an alternative to fluconazole empiric treatment of suspected ICU-acquired candidemia among patients with sepsis: a model simulation
Source: Crit Care. 2009 Jun 19;13(3):R94. doi: 10.1186/cc7924 (PMC2717466; doi:10.1186/cc7924)
Supplement: Additional data file 1 — Table S1 lists the input sources for estimating the prevalence of nosocomial candidemia in critically ill patients. Table S2 lists the input sources for estimating the prevalence of Candida krusei and Candida glabrata as a proportion of all nosocomial candidemia in critically ill patients. [file cc7924-S1.doc]

**Table S1. Input sources for estimating the prevalence of nosocomial candidemia in critically ill patients***

| **Authors** | **Location** | **Publication year** | **Study year(s)** | **% candidemia** |
| --- | --- | --- | --- | --- |
| Alberti [14] | Europe, Canada, Israel | 2002 | 1997-1998 | 9.7 |
| Brun-Buisson [15] | France | 2004 | 2001 | 7.7 |
| Vincent [16] | Europe | 2006 | 2002 | 20.0 |
| Xie [17] | China | 2008 | 2004-2005 | 28.3 |

No US-based studies were identified. Limited to studies in adult ICUs published in year 2000 or later.

**Table S2. Input sources for estimating the prevalence of *C. krusei* and *C. glabrata* as a proportion of all nosocomial candidemia in critically ill patients***

| **Authors** | **Publication year** | **Study year(s)** | **% glabrata** | **% krusei** |
| --- | --- | --- | --- | --- |
| Blumberg [6] | 2001 | 1993-1995 | 19.0% | 2.4% |
| Trick [21] | 2002 | 1989-1999 | 12.0% | 1.0% |
| Swoboda [9] | 2003 | 1990-2002 | 36.1% | 0% |
| Davis [4] | 2007 | 1997-2002 | 13.2% | 0% |
| Shorr [8] | 2007 | 2001-2004 | 18.2% | 0.7% |
| Chow [7] | 2008 | 1995-2005 | 22.6% | 2.1% |

*Studies limited to adult ICUs in the US published in year 2000 or later.
